# Molecular Cloning and Characterization of SaCLCd, SaCLCf, and SaCLCg, Novel Proteins of the Chloride Channel Family (CLC) from the Halophyte *Suaeda altissima* (L.) Pall

**DOI:** 10.3390/plants11030409

**Published:** 2022-02-02

**Authors:** Olga I. Nedelyaeva, Larissa G. Popova, Vadim S. Volkov, Yurii V. Balnokin

**Affiliations:** K.A. Timiryazev Institute of Plant Physiology RAS, 127276 Moscow, Russia; lora_gp@mail.ru (L.G.P.); balnokin@mail.ru (Y.V.B.)

**Keywords:** *Suaeda altissima*, anion transporters, chloride channel family, CLC family, halophytes, molecular cloning, salt tolerance, *SaCLCd*, *SaCLCf*, *SaCLCg*

## Abstract

Coding sequences of the CLC family genes *SaCLCd*, *SaCLCf*, and *SaCLCg*, the putative orthologs of *Arabidopsis thaliana AtCLCd*, *AtCLCf*, and *AtCLCg* genes, were cloned from the euhalophyte *Suaeda altissima* (L.) Pall. The key conserved motifs and glutamates inherent in proteins of the CLC family were identified in SaCLCd, SaCLCf, and SaCLCg amino acid sequences. SaCLCd and SaCLCg were characterized by higher homology to eukaryotic (human) CLCs, while SaCLCf was closer to prokaryotic CLCs. Ion specificities of the SaCLC proteins were studied in complementation assays by heterologous expression of the *SaCLC* genes in the *Saccharomyces cerevisiae GEF1* disrupted strain *Δgef1*. *GEF1* encoded the only CLC family protein, the Cl^−^ transporter Gef1p, in undisrupted strains of this organism. Expression of *SaCLCd* in *Δgef1* cells restored their ability to grow on selective media. The complementation test and the presence of both the “gating” and “proton” conservative glutamates in SaCLCd amino acid sequence and serine specific for Cl^−^ in its selectivity filter suggest that this protein operates as a Cl^−^/H^+^ antiporter. By contrast, expression of *SaCLCf* and *SaCLCg* did not complement the growth defect phenotype of *Δgef1* cells. The selectivity filters of SaCLCf and SaCLCg also contained serine. However, SaCLCf included only the “gating” glutamate, while SaCLCg contained the “proton” glutamate, suggesting that SaCLCf and SaCLCg proteins act as Cl^−^ channels. The *SaCLCd*, *SaCLCf*, and *SaCLCg* genes were shown to be expressed in the roots and leaves of *S. altissima*. In response to addition of NaCl to the growth medium, the relative transcript abundances of all three genes of *S. altissima* increased in the leaves but did not change significantly in the roots. The increase in expression of *SaCLCd*, *SaCLCf,* and *SaCLCg* in the leaves in response to increasing salinity was in line with Cl^−^ accumulation in the leaf cells, indicating the possible participation of SaCLCd, SaCLCf, and SaCLCg proteins in Cl^−^ sequestration in cell organelles. Generally, these results suggest the involvement of SaCLC proteins in the response of *S. altissima* plants to increasing salinity and possible participation in mechanisms underlying salt tolerance.

## 1. Introduction

Soil salinization is a significant problem in agriculture. Salt-affected soils occupy more than 6% of the earth’s land surface (800 million hectares) and, according to various estimates, 20–50% of irrigated land [1,2,3]. The annual losses from salinization in the world currently exceed US $27 billion [4]. The decrease in yield caused by salinity is due to the fact that the vast majority of agricultural crops are salt-sensitive plants, also known as glycophytes [5,6,7,8]. NaCl in soil results in disturbances in plant–water relations and causes Na^+^ and Cl^−^ accumulation up to toxic levels in the cytoplasm [7,9,10,11].

Halophytes are plants of saline habitats that have evolved mechanisms to adequately regulate Na^+^ and Cl^−^ concentrations in cytoplasm and acquire nutrients, in particular nitrate, under soil salinization [12,13,14]. The transport of the cations Na^+^ and K^+^ under salt stress is relatively well studied [6,15,16,17,18,19,20,21]. However, much less is known about the transport of anions. Despite the fact that anion flow into cells is hindered by the plasma membrane electric potential, negative from the cytoplasmic side, Cl^−^ ions at high external concentrations may passively enter the cells and accumulate in the cytoplasm [9,22,23]. Sensitivity of many plants to Cl^−^ is known to be even higher than to Na^+^ [24,25]. To grow under soil salinization, plants, including halophytes, need to maintain relatively low Cl^−^ concentrations in their cytoplasm. For example, the Cl^−^ concentration in the leaf cytoplasm of *Suaeda maritima* growing in 340 mM NaCl was estimated to be approximately 90 mM [26]. One of the main reasons for low NO_3_^−^ availability in plants on salt-affected soils is direct competition of NO_3_^−^ with Cl^−^ for high-affinity anionic transporters [27,28,29,30]. However, nitrate deficiency in halophytes under chloride salinity is less pronounced than in glycophytes [28,29,30]. The ion-transporting proteins of halophytes have been assumed to differ in primary structure, and accordingly in physicochemical properties, from their orthologs in glycophytes [7,29,30,31]. However, researchers have paid little attention to anion transporters despite the supposedly higher efficiency of nitrate transporters of halophytes compared to their orthologs from glycophytes. There are abundant published data on the physiology of salt tolerance in glycophytes but practically no information on the genes encoding anion-transporting proteins of halophytes, which provide absorption, delivery to cells and intracellular regulation of anions, even under strong salinization.

In glycophytes, anion channels and transporters from the CLC (chloride channel) family play key roles in anionic homeostasis, salinity tolerance, and nitrogen nutrition [32,33,34]. CLC proteins are found in representatives of all kingdoms [35,36,37,38]. In addition to chloride channels, this family includes anion/proton exchangers (Cl^−^/H^+^ and NO_3_^−^/H^+^ antiporters) [38]. Contrary to the situation in glycophytes, CLC channels have hardly been studied in halophytes.

In plants, CLC proteins are localized mainly in endomembranes, where they perform many different functions, such as carrying out electrogenic transport of NO_3_^−^ from cytosol into vacuoles [39,40], regulating cytoplasmic concentrations of NO_3_^−^ [41], and participating in acidification of organelles and assumedly in the regulation of their transmembrane electric potential [38]. By analogy with mammalian CLCs, it can be suggested that due to neutralization of positive charges accumulating in organelles as a result of the operation of V-type H^+^-ATPase, the anion/H^+^ exchange executed by plant CLCs promotes compartmentalization of anions in organelles and converts electrical potential into ΔpH. The latter can be subsequently used by secondary transporters as an energy source for transport of diverse substances across the organellar membranes [42]. Cl^−^ transporters are also involved in vesicular trafficking [43,44,45].

Seven genes of the CLC family have been cloned from *A. thaliana*, namely AtCLCa–e. While the functions and physiological roles of their products have been extensively investigated [32,33,34,46], the halophyte orthologs of CLC family proteins remain barely studied. The molecular cloning and functional characterization of proteins from halophytes are important for elucidating the mechanisms underlying plant salt tolerance and improving crop resistance to soil salinity by genetic manipulations [47,48,49,50].

Recently, we cloned *SaCLCa1* and *SaCLCc1*, the putative orthologs of *AtCLCa* and *AtCLCc* encoding NO_3_^−^/H^+^ and Cl^−^/H^+^ antiporters of *A. thaliana*, respectively, from the euhalophyte *Suaeda altissima* [51,52]. In the present work, we describe the cloning of other genes of the CLC family from *S. altissima*, namely *SaCLCd*, *SaCLCf*, and *SaCLCg*, the putative orthologs of *AtCLCd*, *AtCLCf*, and *AtCLCg*, and investigate anion selectivity of the encoded proteins. The anion selectivity of SaCLCd, SaCLCf, and SaCLCg was examined in complementation assays by heterologous expression of their genes in the *Saccharomyces cerevisiae GEF1* disruption mutant Δ*gef1*. *GEF1* is the only gene from the CLC family in *S. cerevisiae*, and the protein Gef1p is characterized by Cl^−^ specificity [53]. Relative *SaCLCd*, *SaCLCf*, and *SaCLCg* transcript levels as well as Cl^−^ content in organs and their biomass were also measured for *S. altissima* plants grown at various NaCl concentrations.

## 2. Materials and Methods

### 2.1. Plant Material

Seeds of *S. altissima* (L.) Pall. were collected from plants growing in the wild on the shores of Lake Elton, a salt lake located in Russia (Volgograd region). The seeds were germinated in wet sand at 21–23 °C. After three weeks, the seedlings were transplanted into a 3 L glass container (4 plants per container) on an aerated Robinson and Downton [54] nutrient solution, supplemented with 250, 500, and 750 mM NaCl or without salt. Plants were then grown in a growth chamber under controlled environmental conditions in water culture at 24 °C and air relative humidity of 60–70%. The plants were illuminated with high-pressure sodium lamps DNaZ_400 “Reflux” (“Minimax”, Saint Petersburg, Russia) with a photoperiod of 16 h/8 h (day/night) and a light intensity of 300 μmol photons/(m^2^·s). Plants that were 45 days old were used in the experiments. For total RNA extraction, leaves and roots of *S. altissima* were sampled (approximately 1 g fresh weight of each sample) and frozen in liquid nitrogen for further use.

### 2.2. Total RNA Extraction and First-Strand cDNA Synthesis

Total RNA samples from *S. altissima* organs were obtained by the hot phenol procedure of Yourieva et al. [55] and used as templates for first-strand cDNA synthesis. For the amplification of 3′- and 5′-ends of *CLC* transcript sequences using Step-Out RACE technology, synthesis of first-strand cDNA was carried out with Mint reverse transcriptase (“Evrogen”, Moscow, Russia) according to the protocol from the manufacturer. For cloning cDNA of CLC family genes and quantitative analysis of *SaCLCd*, *SaCLCf*, and *SaCLCg* transcripts in *S. altissima* organs, synthesis of first-strand cDNA was carried out using total RNA, (dT)_15_ primer, and MMLV reverse transcriptase (“Evrogen”, Moscow, Russia).

### 2.3. Amplification of SaCLCd, SaCLCf, and SaCLCg cDNA Partial Sequences

First, we performed an *in silico* search for the sequences homologous to the *AtCLCd, AtCLCf*, and *AtCLCg* genes in the *de novo* assembled by us transcriptomes of *Suaeda fruticosa* (L.) Forssk, which is a closely related species of *S. altissima* [51,56]. To do this, the contigs of the assembled transcriptomes were translated into amino acid sequences and search for the sequences related to the CLC family proteins was accomplished in the obtained arrays. AtCLCd, AtCLCf, and AtCLCg proteins were used as queries. The primers for amplification of partial cDNA fragments of *S. altissima* homologous genes (Table S1) were then designed using the contigs identified in the assembled *S. fruticosa* transcriptomes and encoding partial sequences of putative chloride channels/transporters. With these primers, the partial cDNAs of *S. altissima* CLC genes were amplified from cDNA template using Encyclo DNA polymerase (“Evrogen”, Moscow, Russia) and sequenced.

### 2.4. Cloning of the Full-Length SaCLCd, SaCLCf, and SaCLCg cDNA Sequences

Based on the partial *SaCLCd*, *SaCLCf*, and *SaCLCg* sequences obtained, the forward and reverse primer sets were designed for amplification of the 3′- and 5′-end fragments (Table S1). With these primers, we amplified the 3′- and 5′-end fragments of *SaCLCd*, *SaCLCf*, and *SaCLCg* (~1000–1500 bp) by 3′- and 5′-rapid amplification of cDNA ends (3′- and 5′-RACE) using the Step-Out RACE technology and cloned them into vector pAL2-T (“Evrogen”, Moscow, Russia). Cloned 3′- and 5′-ends fragments of *SaCLCd*, *SaCLCf*, and *SaCLCg* cDNA were then sequenced. Partial sequences (central fragments, the 3′- and 5′-end fragments of *SaCLCd*, *SaCLCf*, and *SaCLCg*) were then combined *in silico*, and the resulting complete coding sequences for *SaCLCd, SaCLCf,* and *SaCLCg* contained open reading frames (ORFs) for proteins of 793, 587, and 776 amino acids (aa), respectively. Experimentally, the full-size *SaCLCd*, *SaCLCf*, and *SaCLCg* cDNA sequences were amplified with a CloneAmpPCR PreMix kit (“TaKaRa”/Takara Bio Inc., Shiga, Japan; cat # 638916) using pairs of the forward and reverse primers (Table S1) and total first-strand cDNA as a template. The amplified *SaCLCd*, *SaCLCf*, and *SaCLCg* cDNAs were cloned into shuttle vector pMB1, which are designed for expression of proteins in yeast cells [57], under the control of the strong constitutive promoter *GPD1*. A linear form of pMB1 was amplified using the pair of primers (Table S1). The recombinant plasmids pMB1–*SaCLCd*, pMB1–*SaCLCf*, and pMB1–*SaCLCg* were obtained by fusion of *SaCLCd*, *SaCLCf*, and *SaCLCg* cDNAs and the linear form of pMB1 using a Gibson Assembly Cloning kit (“New England Biolabs”, Ipswich, MA, USA). The cloned *SaCLCd, SaCLCf,* and *SaCLCg* were sequenced, and the obtained sequences were deposited in GenBank.

### 2.5. Heterologous Expression of the SaCLCd, SaCLCf, and SaCLCg Genes in Δgef1 Yeast Mutant

*S. cerevisiae* mutant strain *Δgef1* that was created by us earlier [51,52] was transformed with constructs pMB1–*SaCLCd*, pMB1–*SaCLCf*, and pMB1–*SaCLCg* using the lithium protocol [58]. To explore the growth characteristics of the mutant strain Δ*gef1* and the transformants, yeast cells were plated on a number of agarized (2%) selective media described in [59], namely (1) rich YPD medium consisting of 1% yeast extract, 2% peptone, and 2% dextrose (as a fermentable carbon source); (2) rich YPEG medium consisting of 1% yeast extract, 2% peptone, 2% ethanol, and 2% glycerol (as a nonfermentable carbon source); (3) minimal synthetic medium SD [60] supplemented with 2% dextrose and buffered with 50 mM Mes-Tris, pH 7.0; and (4) minimal synthetic medium SR supplemented with 2% raffinose as a nonfermentable carbon source and buffered with 50 mM Mes-Tris, pH 7.0. Yeast cells were left to grow on the selective media for two days (YPD), three days (YPD, YPEG, and SD) or four days (SR) at 28 °C. To study the effect of Mn^2+^ on yeast cell growth, MnCl_2_ or MnSO_4_ were added to the media at final concentrations of 2 or 3 mM. To set up iron deficiency, ferrosin, which is an iron chelator, was added to the media at a final concentration of 1 mM.

### 2.6. Quantitative Analysis of SaCLCd, SaCLCf, and SaCLCg Transcripts in S. altissima Organs 

The cDNA templates for *SaCLCd*, *SaCLCf*, and *SaCLCg* fragment amplification were synthesized on the templates of total RNAs isolated from roots and leaves of *S. altissima* plants grown on nutrient media with various NaCl concentrations. Quantitative analysis of *SaCLCd*, *SaCLCf*, and *SaCLCg* transcripts was performed by the qRT-PCR method using a LightCycler^®^ 96 system (Roche Diagnostics Corporation, Indianopolis, IN, USA). A reaction mixture with intercalating dye SYBR Green I (“Evrogen”, Moscow, Russia) was used. To amplify the *SaCLCd*, *SaCLCf*, and *SaCLCg* fragments, the pairs of primers were used (Table S1). Target gene mRNA expression levels were normalized for the *S. altissima* actin gene *SaAct7* (GenBank, acc. no. MK615596.1) and the elongation factor 1 alpha gene *SaeEF1alpha* (GenBank, acc. no. MN076325.1). To amplify the *SaAct7* and *SaeEF1alpha* fragments, the primer pairs were used (Table S1). Results were based on three to five biological replicates. The results obtained were processed by LightCycler 96SW 1.1 software. The expression of the selected reference genes was quite stable with fold changes not exceeding 0.4 under the chosen experimental conditions.

### 2.7. Primer Design

Primers for qPCR-RT experiments were designed by Light Cycler96 Probe Design software (https://lifescience.roche.com/, accessed on 15 December 2021). Other primers were designed using Oligo 7 software (https://www.oligo.net/, accessed on 15 December 2021) or Primer Blast software (https://www.ncbi.nlm.nih.gov/tools/primer-blast/, accessed on 15 December 2021). The primers are listed in Table S1.

### 2.8. Bioinformatic Analysis of Amino Acid Sequences

Multiple alignment of amino acid sequences of CLC proteins was performed by MAFFT software using on-line service (https://www.ebi.ac.uk/Tools/msa/mafft/, accessed on 15 December 2021). The phylogenetic tree of plant CLC family proteins was created by Molecular Evolutionary Genetic Analysis (MEGA) 11 software (https://www.megasoftware.net/, accessed on 15 December 2021) using the maximum likelihood method based on the Jones–Taylor–Thornton model [61] (1000 bootstrap replication performed). Protein topology was predicted by MEMSAT-SVM software (http://bioinf.cs.ucl.ac.uk/software_downloads/memsat/, accessed on 15 December 2021). Intracellular localization of the proteins was predicted with the DeepLoc 1.0 software’s eukaryotic protein subcellular localization predictor (http://www.cbs.dtu.dk/services/DeepLoc-1.0/index.php, accessed on 15 December 2021).

### 2.9. Determination of Chloride Content in S. altissima Organs

Water extracts from *S. altissima* roots and leaves were prepared by incubating samples that were dried for 1 day at 90 °C and then ground in boiling deionized water for 10 min. Concentrations of Cl^−^ in the extracts were determined by titration with Hg^2+^ using a Top Buret H digital burette (Eppendorf, Wesseling-Berzdorf, Germany).

### 2.10. Statistical Analysis

Statistical analysis of the data was made by one-way analysis of variance (ANOVA). A *p*-value < 0.05 was considered to be statistically significant. * *p* ≤ 0.05; ** *p* ≤ 0.01; *** *p* ≤ 0.001. Standard deviations are given. Correlation coefficients were calculated in the Excel program.

## 3. Results

Coding nucleotide sequences of *SaCLCd*, *SaCLCf*, and *SaCLCg*, genes from the halophyte *S. altissima*, were determined based on the putative similarity of these genes to homologous genes from the halophyte *S. fruticosa*. As a result of *in silico* searches of sequences related to the *CLC* family in the *de novo* assembled transcriptome of *S. fruticosa* [51,56], the contigs containing the partial coding regions of three sequences homologous to the *A. thaliana CLC* genes were designated by us as *SfCLCd*, *SfCLCf*, and *SfCLCg.* The contigs from *S. fruticosa* served as a base for identification of the full-size coding sequences of the target *S. altissima* genes by a rapid amplification of 3´- and 5´-cDNA ends. The cDNAs of the *SaCLCd*, *SaCLCf*, and *SaCLCg* genes thus obtained were then cloned and sequenced. The cloned cDNAs of *SaCLCd* (GenBank, acc. no. OK626332), *SaCLCf* (GenBank, acc. no. OK626333), and *SaCLCg* (GenBank, acc. no. OK626334) genes contained open reading frames (ORFs) encoding polypeptides consisting of 793, 587, and 776 amino acids, with calculated molecular masses of 87.6, 62.3, and 85.8 kDa, respectively. SaCLCd and SaCLCg were of molecular masses close to those of most plant and animal CLC proteins [37,62,63,64,65]. SaCLCf was noticeably smaller than the other two proteins. It should be noted that similar but smaller CLC proteins have been found in other plants. The *AtCLCf* gene encodes two forms of the AtCLCf protein, one with molecular mass of 83.5 kDa (781 a.a., At1g55620.2) and a shorter one with molecular mass of 62.5 kDa (586 a.a., At1g55620.1) [37] (Figure S1). Moreover, shortened *CLCf* transcripts with corresponding shortened proteins were revealed in transcriptomes of grape (*Vitis vinifera*) (GenBank: NP_001268117.1), pistachio (*Pistacia vera*) (GenBank: XP_031257549.1), and alfalfa (*Medicago truncatula*) (GenBank: KEH32883.1).

**Figure 1:**
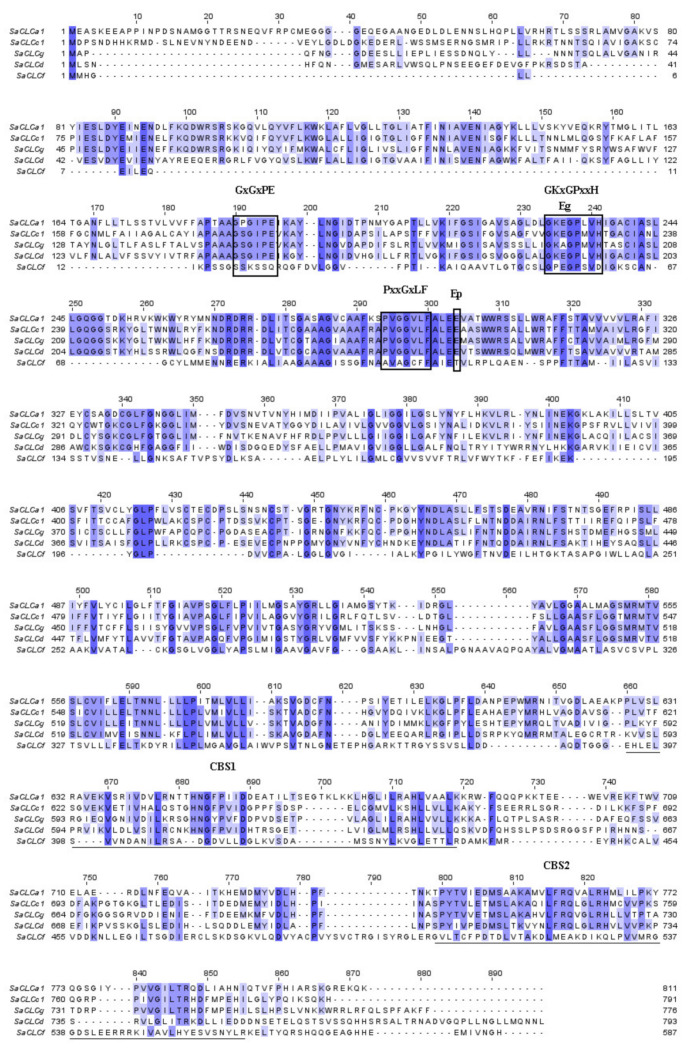
Alignment of the amino acid sequences of CLC proteins from *S. altissima*: SaCLCa1 (GenBank, acc. no. ANG09048.1), SaCLCc1 (GenBank, acc. no. AVQ93350.1), SaCLCd (GenBank, acc. no. OK626332), SaCLCf (GenBank, acc. no. OK626333), and SaCLCg (GenBank, acc. no. OK626334). The alignment was performed in the MAFFT program and visualized in Jalview 2.11.1.4 program [70]. The conserved amino acid motifs (GxGxPE, GKxGPxxH and PxxGxLF) are framed. GxGxPE motif is a selective filter. Eg and Ep are the key glutamates of the CLC family proteins. The intensity of staining for amino acid residues depicts the degree of their identity (percentage identity). CBS1 and CBS2 domains are underlined.

Each of the three proteins identified in *S. altissima* contained three conserved motifs (Figure 1) that are a distinctive feature of all CLC proteins. In the amino acid sequences of the SaCLC proteins, the motifs occupied the positions given in Table S2. The motifs of two proteins, SaCLCd and SaCLCg, were found to match the next sequences, namely (1) GxGxPE, (2) GKxGPxxH, and (3) PxxGxLF revealed earlier in *A. thaliana* [32]. The three homologous motifs identified in SaCLCf, namely (1) SSKSSQ, (2) GPEGPSVD, and (3) AVAGCFF, differed from those of SaCLCd and SaCLCg and were almost identical to the motifs of AtCLCf (Figure S1).

According to [66], the conserved motifs in CLC proteins are involved in the formation of the anion-conducting pathway through membrane, in determination of channel ionic selectivity, and in gating of anion-conducting pathway. The motif GSGIPE and its putative homolog SSKSSQ (Figure 1) are functional as selectivity filters [66,67]. The amino acid occupying the second position in the motifs has been shown to be responsible for anionic specificity of the CLC protein, namely proline (P) for NO_3_^−^ and serine (S) for Cl^−^ [68,69]. The GSGIPE (SSKSSQ in SaCLCf) motif of the *S. altissima* CLC proteins identified in the current study included serine in the second position, thus suggesting involvement of these proteins in chloride transport.

Two conserved glutamates, Eg (“gating” glutamate) and Ep (“proton” glutamate), play key roles in the functioning of anion/H^+^ antiporters of the CLC family [36,71]. Eg participates in the gating of the transmembrane anion path, whereas Ep is necessary for H^+^ translocation [72]. We found both conserved glutamates inherent in CLC anion/H^+^ antiporters in only the SaCLCd amino acid sequence. Only “gating” glutamate (Eg) was found in SaCLCf, and only “proton” glutamate (Ep) was found in SaCLCg (Table S2). This suggests that the ion transport mechanisms differ from anion/proton antiport for SaCLCf and SaCLCg.

Like other CLCs, SaCLCd, SaCLCf, and SaCLCg contain the regulatory cystathionine beta synthase (CBS) domains CBS1 and CBS2 in the hydrophilic region at the C-terminus (Figure 1; Table S2).

According to the topology models predicted by the MEMSAT-SVM software, SaCLCd, SaCLCf, and SaCLCg are integral membrane proteins. They form 11, 7, and 9 transmembrane domains, respectively, with the N- and C-ends of the protein located on opposite sides of the membrane.

**Figure 2:**
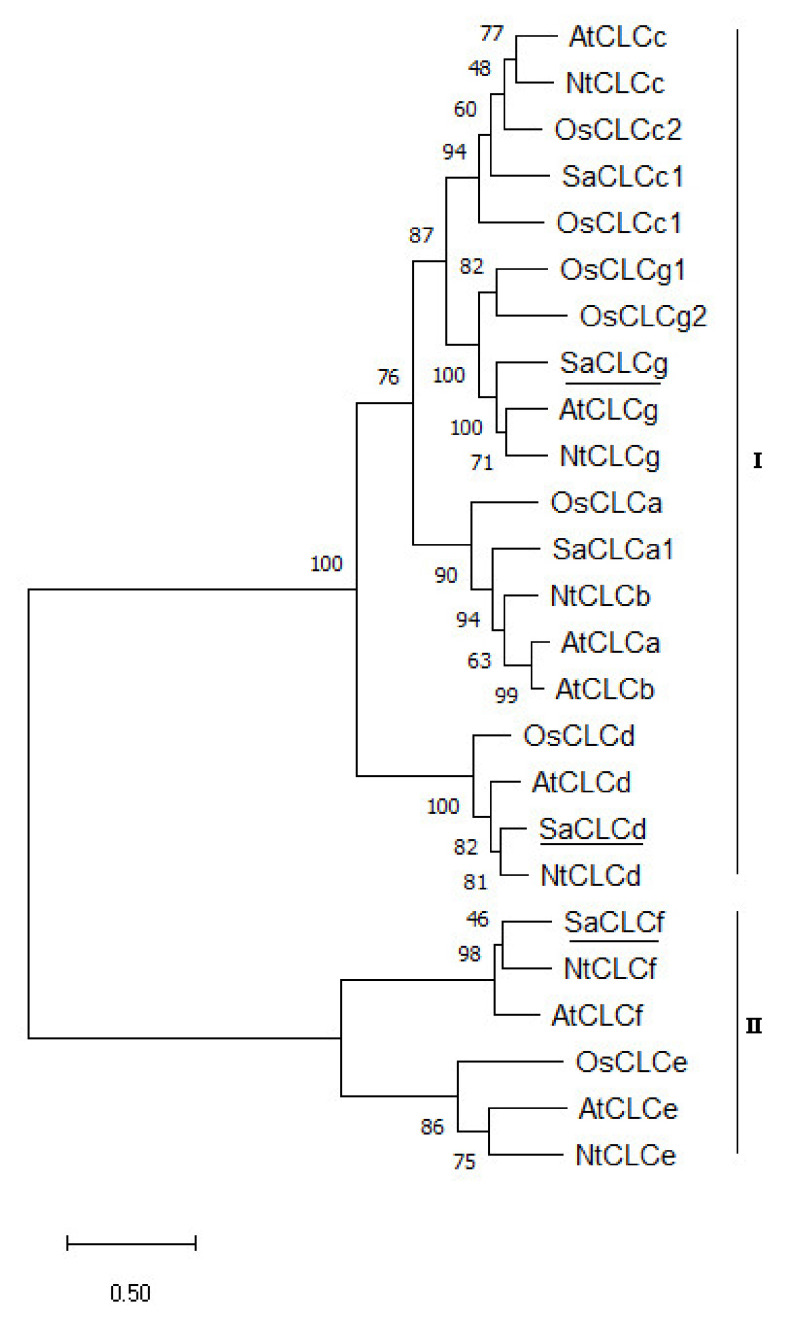
Phylogenetic tree of CLC family proteins of *A. thaliana*, *O. sativa*, *N. tabacum,* and *S. altissima*. AtCLCa (NP_198905.1), AtCLCb (NP_189353.1), AtCLCc (NP_199800.1), AtCLCd (NP_197996.1), AtCLCe (NP_567985.1), AtCLCf (NP_564698.1), AtCLCg (NP_198313.2), OsCLC1 (XP_015633162.1), OsCLC2 (XP_015622009.1), OsCLC3 (XP_015626588.1), OsCLC4 (AAO19370.1), OsCLC5 (XP_015636607.1), OsCLC6 (XP_015650515.1), OsCLC7 (XP_015620662.1), NtCLCc (NP_001312418.1), NtCLCb (NP_001312163.1), NtCLCd (XP_016512457.1), NtCLCe (XP_016461326.1), NtCLCf (XP_009787963.1), NtCLCg (XP_016468444.1), SaCLCa1 (ANG09048.1), SaCLCc1 (AVQ93350.1), SaCLCd (OK626332), SaCLCf (OK626333), and SaCLCg (OK626334). All protein sequences were taken from the protein database (NCBI). Subgroup I is the “eukaryotic” branch, and subgroup II is the “prokaryotic” branch. The phylogenetic tree was built in the MEGA 11 using the maximum likelihood method based on the Jones–Taylor–Thornton model. The number of bootstrap replicates was 1000; the values of bootstrap support are indicated near the nodes. Scale: 0.5 substitutions per site.

Phylogenetic analysis (Figure 2) revealed a similarity between SaCLCd, SaCLCf, and SaCLCg and CLC family representatives from other plants. Therefore, we named the cloned genes of *S. altissima* CLC proteins based on their similarity to *A. thaliana* CLC proteins characterized earlier.

The known CLCs can be divided into two subfamilies. One of them is characterized by a higher homology to eukaryotic (mostly human) CLCs; the representatives of the other are closer to prokaryotic CLCs [37,45]. Accordingly, in our cladogram of plant CLCs, the proteins were also divided into two clusters (Figure 2). We found SaCLCd and SaCLCg in the first “eukaryotic” cluster and SaCLCf in the second “prokaryotic” one. Interestingly, according to predictions obtained with the DeepLoc 1.0 software, “eukaryotic” SaCLCd and SaCLCg as well as “prokaryotic” SaCLCf were more likely to be localized to the vacuolar membrane (P_SaCLCd_ = 0.59; P_SaCLCf_ = 0.34; P_SaCLCg_ = 0.64) than to the plasma membrane (P_SaCLCd_ = 0.39; P_SaCLCf_ = 0.18; P_SaCLCg_ = 0.32). However, the “prokaryotic” SaCLCf gave indications of localization in mitochondria (P_SaCLCf_ = 0.19), endoplasmic reticulum (P_SaCLCf_ = 0.11), plastids (P_SaCLCf_ = 0.11), or Golgi network (P_SaCLCf_ = 0.05), which could be linked to the symbiogenetic (or partially symbiogenetic) origin of the organelles.

To elucidate the transport functions of SaCLCd, SaCLCf, and SaCLCg proteins, we used the previously generated [51,52] knockout mutant strain *Δgef1* of *S. cerevisiae*. Such mutants have successfully been used earlier for clarifying anion selectivity of CLC proteins from diverse organisms [24,59,73,74,75,76]. The Gef1p protein, encoded by *GEF1*, transports chloride and is the single member of the chloride channel family in *S. cerevisiae*. Knockout mutation of the *GEF1* gene caused disturbances in a range of cellular processes and led to corresponding phenotypic manifestations [53,59]. The growth of *Δgef1* was suppressed on rich media containing nonfermentable carbon sources (glycerol, ethanol, acetate, lactate, and raffinose) and iron in reduced concentrations and on media with fermentable carbon sources (glucose, fructose, and mannose) at pH 7.0. As the solubility of iron salts decreases upon moderate alkalization, the availability of iron for yeast cells was also lowered to pH ≥ 7.0. Suppressed growth of Δ*gef1* was also found on media containing cations (Li^+^, Mn^2+^, Ca^2+^, Mg^2+^, and tetramethylammonium^+^) in toxic concentrations.

**Figure 3:**
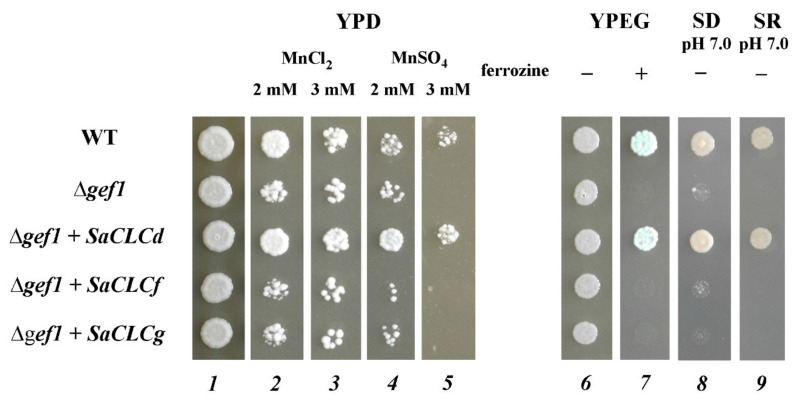
The growth of the yeast mutant Δ*gef1* transformed with *SaCLCd*, *SaCLCf*, and *SaCLCg* genes. Controls: wild-type W3031A and the mutant Δ*gef1* transformed with vector pMB1. Selective media: lanes 1—YPD (YPD medium: 1% yeast extract, 2% peptone, and 2% dextrose; 2 days of cells growth); 2—YPD + 2 mM MnCl_2_ (2 days); 3—YPD + 3 mM MnCl_2_ (2 days); 4—YPD + 2 mM MnSO_4_ (3 days); 5—YPD + 3 mM MnSO_4_ (3 days); 6—YPEG (rich YPEG medium: 1% yeast extract, 2% peptone, 2% ethanol, and 2% glycerol; 3 days); 7—YPEG + 1 mM ferrozine (Fe^2+^ chelator) (3 days); 8—SD, pH 7.0 (minimal synthetic medium [60] supplemented with 2% dextrose; 3 days); 9—SR, pH 7.0 (minimal synthetic medium supplemented with 2% raffinose; 4 days). Approximately 10^5^ of the yeast cells were plated on selective media.

We transformed the yeast mutant strain Δ*gef1* by the constructs pMB1–*SaCLCd*, pMB1–*SaCLCf*, and pMB1–*SaCLCg* created on the basis of the shuttle vector pMB1 and containing sequences *SaCLCd*, *SaCLCf*, and *SaCLCg* under control of the strong constitutive *GPD1* promoter. To determine the phenotype of the transformants, the strains obtained were plated on the agarized selective diagnostic media described above (Figure 3). According to Gaxiola et al. [59], Δ*gef1* cells fail to grow on Fe-deficient medium YPEG with nonfermentable carbon sources and on both SD and SR synthetic media at pH 7.0. In our experiments, growth of Δ*gef1* cells failed under an Fe-deficient setup when Fe chelator ferrozine was added to YPEG medium with ethanol and glycerol as carbon sources. The same was the case when the pH was adjusted to 7.0 for SD and SR media. The growth of the mutant strain Δ*gef1* on rich YPD medium was also inhibited by Mn^2+^ ions at concentrations of 2 or 3 mM (Figure 3).

Expression of *SaCLCf* or *SaCLCg* did not restore the growth of Δ*gef1* colonies on the selective media. However, growth restoration of Δ*gef1* occurred when the mutant strain was transformed with the construct pMB1–*SaCLCd* (Figure 3), indicating the recovery of Cl^−^/H^+^ exchanger function in the mutant cells.

**Figure 4:**
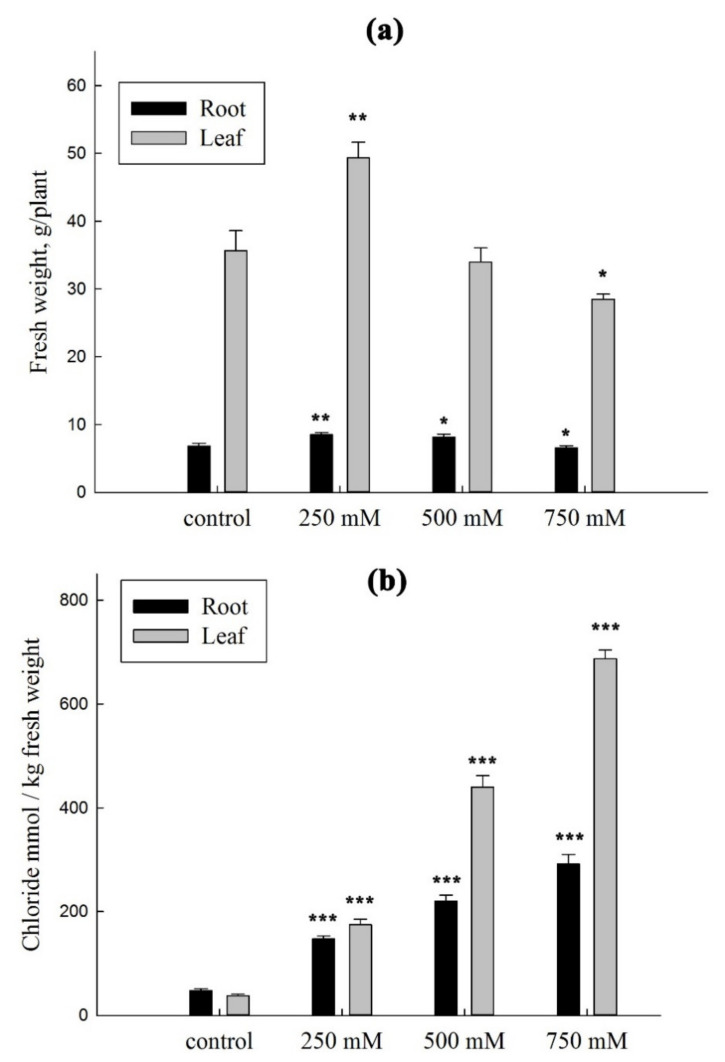
Fresh weights (**a**) and chloride contents (**b**) of leaves and roots of *S. altissima* grown at various NaCl concentrations in the nutrient medium. A *p*-value < 0.05 was considered to be statistically significant. * *p* ≤ 0.05; ** *p* ≤ 0.01; *** *p* ≤ 0.001. Standard deviations are given.

To gain further insight into the putative physiological functions of SaCLCd, SaCLCf, and SaCLCg, we investigated expression of genes encoding these proteins in the roots and leaves of *S. altissima* plants grown under increasing NaCl concentrations in the nutrient solution. We also determined the growth characteristics of *S. altissima* plants and the contents of chloride in *S. altissima* organs under these conditions. The growth of *S. altissima* was stimulated for both roots and shoots at 250 mM NaCl and stimulated for roots even at 500 mM NaCl, while inhibition (compared to 0 mM NaCl) only started at 750 mM NaCl (Figure 4a). Chloride was accumulated in *S. altissima* organs under salinity. The contents of chloride in the root and leaf tissues of *S. altissima* linearly increased with increasing NaCl concentration in the nutrient solution, and the increase was more pronounced in the leaves than in the roots (Figure 4b). With NaCl addition, concentration of chloride in the leaves were only slightly lower than that in the nutrient solution and much higher than that in the roots. At 750 mM NaCl in the nutrient solution, the concentration of chloride in the roots was less than half that in the leaves and in the nutrient solution.

**Figure 5:**
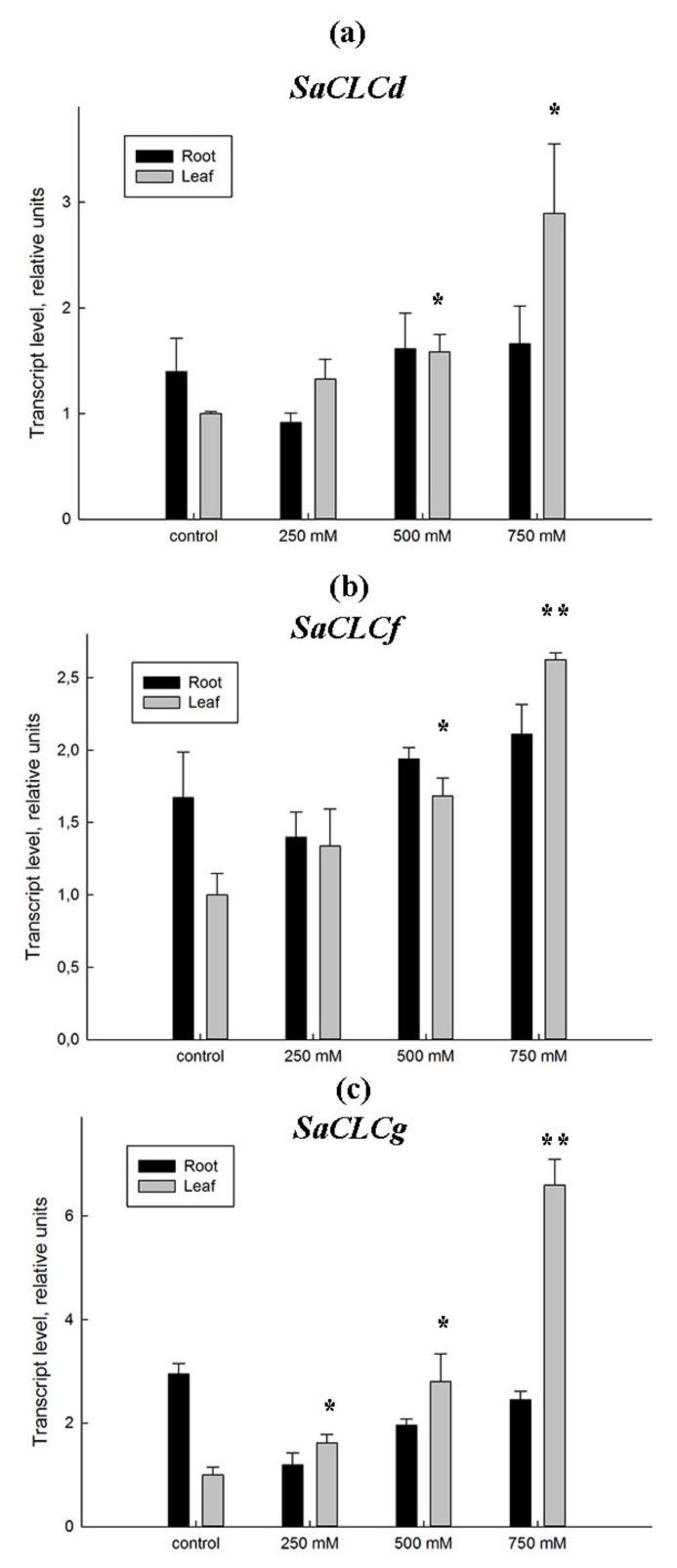
Relative abundance of the *SaCLCd* (**a**), *SaCLCf* (**b**), and *SaCLCg* (**c**) transcripts in the roots (dark bars) and leaves (light bars) of *S. altissima* plants grown at various NaCl concentrations in the plant growth medium. The actin gene *SaAct7* was used as internal reference gene. Similar results were obtained with *SaAct7* and *SaeEF1alpha* as reference genes, so the data are presented for the actin gene only. Data shown are means ± SD from three independent experiments. The results were deduced from three biological replicates, and each of them were performed in three analytical replicates. A *p*-value < 0.05 was considered to be statistically significant. * *p* ≤ 0.05; ** *p* ≤ 0.01.

The expression of *SaCLCd*, *SaCLCf*, and *SaCLCg* genes showed different patterns for roots and leaves of the euhalophyte under increasing salinity (Figure 5), although changes in the transcript levels were similar for all three genes. In the leaves, the relative quantity of *SaCLCd*, *SaCLCf*, and *SaCLCg* transcripts grew linearly as the salt concentration in the medium increased. Correlation coefficients (R^2^) between chloride content and the level of expression of *SaCLCd*, *SaCLCf*, and *SaCLCg* genes were 0.946, 0.975 and 0.951, respectively. In the roots, the relative quantity of the *CLC* transcripts did not change significantly with increasing salinity. The minor changes observed in the expression of *CLCs* genes in the roots were in good agreement with smooth and relatively moderate changes in the Cl^−^ content in this organ (Figure 4b). In the leaves, a significant accumulation of Cl^−^ ions, observed from the elevation of NaCl concentration in the medium, corresponded to a noticeable increase in the expression of *SaCLCd*, *SaCLCf*, and *SaCLCg*, indicating the possible participation of CLC proteins that were presumably residing in the tonoplast and mediating vacuolar ion accumulation.

## 4. Discussion

In this work, we cloned *SaCLCd*, *SaCLCf*, and *SaCLCg* genes of CLC family, the putative orthologs of *AtCLCd*, *AtCLCf*, and *AtCLCg* genes, from the euhalophyte *S. altissima*. The primary structure, membrane topology, and phylogenetic analysis of the cloned genes confirmed that they belong to the chloride channel family and have common properties both with representatives of this family from other plant species and with each other. At the same time, the three cloned *S. altissima* CLC proteins displayed some differences.

*SaCLCd*, like the homologous *A. thaliana* gene *AtCLCd*, belongs to the “eukaryotic” subfamily of *CLC* family genes (Figure 2) [37,45,52]. Amino acid sequences of both SaCLCd and AtCLCd contained the two conserved glutamates Eg and Ep (Figure 1), a hallmark of the “eukaryotic” subfamily of CLC proteins [71,77]. Serine in the second position of GSGxPE motif (Figure 1) indicates a preference for Cl^−^ over NO_3_^−^ in anionic specificity of both these proteins [45,59]. Bioinformatic predictions located the corresponding protein for *SaCLCd* to tonoplast or plasma membrane with combined probability of 0.98.

In complementation analyses, AtCLCd is often used as a positive control for heterologous expression of CLC genes from diverse organisms in the *Saccharomyces cerevisiae GEF1* disrupted strain Δ*gef1*. Expression of *AtCLCd* in the Δ*gef1* strain invariably rescues the growth defect phenotype of the latter [37,52,59,78]. Like *AtCLCd*, the expression of *SaCLCd* gene also complemented the growth defect phenotype of the Δ*gef1* yeast mutant. The cells of Δ*gef1* transformed with the construct pMB1–*SaCLCd*, in contrast to Δ*gef1* cells transformed with empty vector pMB1, demonstrated indistinguishable growth from that of WT *S*. *cerevisiae* cells on selective media (Figure 3). Altogether, these results indicate that SaCLCd operates as an anion/H^+^ exchanger rather than an anion channel.

*SaCLCf* belongs to the “prokaryotic” subfamily of *CLC* family genes (Figure 2). AtCLCf, the homolog of SaCLCf protein, is currently the least studied protein of the AtCLC family, and its subcellular localization, transport functions, and physiological role are not yet fully clarified. The available data concerning its localization and functions are contradictory. For yeast grown on selective media [59], complementation of the growth defect phenotype of Δ*gef1* was observed in the mutant cells expressing AtCLCf [37]. However, Lv and coworkers [78] did not obtain complementation of the *Δgef1* mutant phenotype in response to the expression of this gene. According to [37], AtCLCf mainly resides in *cis*-Golgi compartments and, to a lesser extent, in the *trans*-Golgi network. However, a recent work [45] demonstrated that the relation of AtCLCf is predominantly to the *trans*-Golgi network/early endosomes (TGN/EE), where it colocalizes with V-ATPase (VHA-a1) as well as AtCLCd. The authors suggested that AtCLCd and AtCLCf transfer Cl^−^ into the endosomal lumen and, together with V-ATPase, are likely involved in regulation of pH and chloride concentration in the lumen of the *trans*-Golgi network and in late endosomes. Our bioinformatic predictions located the homolog of AtCLCf protein, SaCLCf, to tonoplast or plasma membrane with a combined probability of 0.52. Arguably, SaCLCf is equally likely to be located in the Golgi network or endosome membranes, similar to the AtCLCf protein.

We failed to complement the growth defect phenotype of the yeast mutant strain *Δgef1* by expressing SaCLCf in its cells (Figure 3). Given the presence of serine in the second position of the SaCLCf conserved motif SSKSSQ (Figure 1), which indicates chloride specificity of this protein [66,67], the complementation failure could be because SaCLCf functions as a chloride channel and not as a Cl^−^/H^+^ antiporter. In line with the result, amino acid sequence of SaCLCf did not contain Ep, the conserved glutamate (Figure 1), playing a key role in the H^+^ transport through membrane [71,77]. In relation to these data, it should be noted that “neutralization” of “proton” glutamate E203 in prokaryotic CLC-ec1 by mutation E270Q abolished H^+^ coupling to Cl^−^ transport [72]. However, the suggestion that CLCfs act as chloride channels did not find support in a recently proposed hypothesis [45]. It was hypothesized that in members of the “prokaryotic” subfamily AtClCe and AtClCf, the function of “proton” glutamate might be performed by a glutamate other than the Ep of the “eukaryotic” CLC subfamily and that these “prokaryotic” representatives of the CLC family are likely antiporters [45]. Generally, the operation mechanism of SaCLCf, like that of AtCLCf, remains to be elucidated.

The third gene *SaCLCg*, the homolog of *AtCLCg*, was a member of the “eukaryotic” subfamily of *S. altissima CLCs*. The presence of serine in the *SaCLCg* protein in the second position of the conserved motif GSGIPE, canonic for the “eukaryotic” CLC subfamily, indicated chloride selectivity of SaCLCg. Expression of *SaCLCg* in cells of the yeast mutant strain *Δgef1* did not complement its growth defect phenotype (Figure 3) like the expression of the *AtCLCg* homolog [78]. This fact, together with the missing “gating” glutamate in its amino acid sequence (Figure 1), indicates that SaCLCg, like its homolog from *Arabidopsis*, is a chloride channel rather than a Cl^−^/H^+^ antiporter. The importance of Eg for functioning “eukaryotic” anion/H^+^ antiporters is highlighted by the fact that mutating the “gating” glutamate E203 to alanine in AtCLCa expressed in *Xenopus* oocytes resulted in uncoupled anion conductance [71]. AtCLCg, most likely a Cl^−^ channel [33,38,71], is expressed in mesophyll, phloem, and hydathode cells of mature leaves as well in root cells; the protein was shown to localize in tonoplast [46,78]. AtCLCg was suggested to be involved in sequestering Cl^−^ ions in vacuoles, phloem recirculation, guttation, and xylem loading, thereby providing tolerance to salt stress [46]. Minimizing xylem loading and subsequent transport of Cl^−^ to the shoot might contribute to salt tolerance by keeping photosynthetic tissues away from Cl^−^ overaccumulation [9]. The euhalophytes have evolved another strategy for growth under salinity. These plants translocate absorbed Na^+^ and Cl^−^ to the shoots and accumulate them preferentially in leaf vacuoles [14,79,80]. This maintains cytoplasmic Na^+^ and Cl^−^ concentrations at nontoxic levels and contributes to water potential gradient setup in the system, namely soil–root–shoot, which promotes continuous water flow in the ascending direction [81]. Greater Na^+^ and Cl^−^ accumulation in *S. altissima* leaves than in the roots and the growth stimulation in response to increase in NaCl concentration in the medium (Figure 4) are in line with patterns of SaCLCd, SaCLCf, and SaCLCg expression in these organs under saline conditions (Figure 5). Results of the study of *SaCLC* gene expression in the roots and leaves of plants grown at different NaCl concentrations in the medium showed that abundance of *SaCLCd*, *SaCLCf*, and *SaCLCg* transcripts did not change significantly in the roots with increasing salinity in the nutrient solution (Figure 5). However, in the leaves, a substantial increase in *SaCLCd*, *SaCLCf*, and *SaCLCg* transcript levels was observed under these conditions, which implies the participation of the proteins encoded by these genes in Cl^−^ accumulation in leaf organelles.

## 5. Conclusions

In this study, we cloned coding sequences of three novel chloride channel family genes *SaCLCd*, *SaCLCf*, and *SaCLCg*, the putative orthologs of *A. thaliana AtCLCd*, *AtCLCf*, and *AtCLCg* genes, from the euhalophyte *S. altissima*. The growth of this euhalophyte was stimulated by 250 mM NaCl, while inhibition only started from 750 mM. This is an extremely unusual feature for most plants, including agricultural ones that are glycophytes. However, global climate change and soil salinization have made us consider the importance of halophytes. In plants, members of the chloride channel family transport Cl^−^ and NO_3_^−^ across membranes of intracellular organelles and account for a number of physiological functions. The results of a complementation assay using yeast expression system as well as bioinformatic analyses of the proteins encoded by these genes indicate that SaCLCd protein is a Cl^−^/H^+^ antiporter, while the SaCLCf and SaCLCg proteins are likely Cl^−^ channels. The results of qRT-PCR analyses showed that expression of all three genes was activated in the leaves with increase in NaCl concentration in the growth medium, suggesting the involvement of the SaCLCd, SaCLCf, and SaCLCg proteins in the response of *S. altissima* to NaCl. All three encoded proteins share common properties with the proteins of CLC family representatives from glycophytes, particularly the presence of conserved motifs, conserved glutamates, and regulatory CBS domains in amino acid sequences. However, differences between the proteins of *S. altissima* and their putative orthologs from glycophytes remain to be elucidated. Future investigations of the proteins, such as studies aimed at revealing their intracellular localization and distribution in whole plant, functional studies employing heterological expression systems, and examination of the physiochemical and structural protein features, could clarify differences between halophyte and glycophyte proteins and give clues to understanding the processes underlying salinity tolerance and ways to improve them by methods of molecular genetics.

## Data Availability

All data included in this study are available upon request by contact with the corresponding author. The cloned *SaCLCd*, *SaCLCf*, and *SaCLCg* cDNA were deposited in GenBank (acc. no. OK626332, OK626333, and OK626334, respectively).

## Supplementary Materials

The following supporting information can be downloaded at: https://www.mdpi.com/article/10.3390/plants11030409/s1, Figure S1: Alignment of the amino acid sequences SaCLCf (GenBank, acc. no. OK626333), AtCLCf_long (GenBank, acc. no. NP_564698.1), and AtCLCf_short (GenBank, acc. no. NP_849813.1) performed in the MAFFT program and visualized in Jalview 2.11.1.4 program [70]. The intensity of the staining of amino acid residues corresponds to the degree of their identity (Percentage Identity), Table S1: List of the primers used in the study, Table S2: Conserved amino acids motifs and residues in SaCLCd, SaCLCf and SaCLCg sequences and their coordinates.

## References

[1] Flowers T.J. (1999). Salinisation and horticultural production. Sci. Hortic..

[2] UNESCO World Water Quality Portal Unesco. https://en.unesco.org/waterqualitymonitor.

[3] Ismail A.M., Horie T. (2017). Genomics, Physiology, and Molecular Breeding Approaches for Improving Salt Tolerance. Annu. Rev. Plant Biol..

[4] Qadir M., Quillérou E., Nangia V., Murtaza G., Singh M., Thomas R.J., Drechsel P., Noble A.D. (2014). Economics of salt-induced land degradation and restoration. Nat. Resour. Forum.

[5] Wang B., Davenport R.J., Volkov V., Amtmann A. (2006). Low unidirectional sodium influx into root cells restricts net sodium accumulation in Thellungiella halophila, a salt-tolerant relative of Arabidopsis thaliana. J. Exp. Bot..

[6] Apse M.P., Blumwald E. (2007). Na^+^ transport in plants. FEBS Lett..

[7] Munns R., Tester M. (2008). Mechanisms of salinity tolerance. Annu. Rev. Plant Biol..

[8] Yamaguchi T., Hamamoto S., Uozumi N. (2013). Sodium transport system in plant cells. Front. Plant Sci..

[9] Teakle N.L., Tyerman S.D. (2010). Mechanisms of Cl^−^ transport contributing to salt tolerance. Plant Cell Environ..

[10] Agarwal P.K., Shukla P.S., Gupta K., Jha B. (2013). Bioengineering for salinity tolerance in plants: State of the art. Mol. Biotechnol..

[11] Mbarki S., Sytar O., Cerda A., Zivcak M., Rastogi A., He X., Zoghlami A., Abdelly C., Brestic M., Kumar V., Wani S.H., Penna S., Tran L.S.P. (2018). Strategies to mitigate the salt stress effects on photosynthetic apparatus and productivity of crop plants. Salinity Responses and Tolerance in Plants.

[12] Flowers T.J., Galal H.K., Bromham L. (2010). Evolution of halophytes: Multiple origins of salt tolerance in land plants. Funct. Plant Biol..

[13] Shabala S. (2013). Learning from halophytes: Physiological basis and strategies to improve abiotic stress tolerance in crops. Ann. Bot..

[14] Flowers T.J., Munns R., Colmer T.D. (2015). Sodium chloride toxicity and the cellular basis of salt tolerance in halophytes. Ann. Bot..

[15] Shi H., Ishitani M., Kim C., Zhu J.K. (2000). The Arabidopsis thaliana salt tolerance gene SOS1 encodes a putative Na^+^/H^+^ antiporter. Proc. Natl. Acad. Sci. USA..

[16] Shi H., Quintero F.J., Pardo J.M., Zhu J.K. (2002). The putative plasma membrane Na^+^/H^+^ antiporter SOS1 controls long-distance NA+ transport in plants. Plant Cell.

[17] Tester M., Davenport R. (2003). Na^+^ tolerance and Na^+^ transport in higher plants. Ann. Bot..

[18] Davenport R.J., Muñoz-Mayor A., Jha D., Essah P.A., Rus A., Tester M. (2007). The Na^+^ transporter AtHKT1;1 controls retrieval of Na^+^ from the xylem in *Arabidopsis*. Plant Cell Environ..

[19] James R.A., Blake C., Zwart A.B., Hare R.A., Rathjen A.J., Munns R. (2012). Impact of ancestral wheat sodium exclusion genes Nax1 and Nax2 on grain yield of durum wheat on saline soils. Funct. Plant Biol..

[20] Reguera M., Bassil E., Blumwald E. (2014). Intracellular NHX-Type cation/H^+^ antiporters in plants. Mol. Plant.

[21] Wang Z., Hong Y., Zhu G., Li Y., Niu Q., Yao J., Hua K., Bai J., Zhu Y., Shi H. (2020). Loss of salt tolerance during tomato domestication conferred by variation in a Na^+^/K^+^ transporter. EMBO J..

[22] Pantoja O., Dainty J., Blumwald E. (1992). Cytoplasmic Chloride Regulates Cation Channels in the Vacuolar Membrane of Plant Cells. J. Membr. Biol..

[23] White P.J., Broadley M.R. (2001). Chloride in soils and its uptake and movement within the plant: A review. Ann. Bot..

[24] Wei P., Wang L., Liu A., Yu B., Lam H.M. (2016). GmCLC1 confers enhanced salt tolerance through regulating chloride accumulation in soybean. Front. Plant Sci..

[25] Li B., Tester M., Gilliham M. (2017). Chloride on the move. Trends Plant Sci..

[26] Flowers T.J., Yeo A.R., Baker D., Hall J. (1988). Ion relation of salt tolerance. Solute Transport in Plant Cells and Tissues.

[27] Rubinigg M., Posthumus F., Ferschke M., Elzenga J.T.M., Stulen I. (2003). Effects of NaCl salinity on 15N-nitrate fluxes and specific root length in the halophyte Plantago maritima L. Plant Soil.

[28] Song J., Ding X., Feng G., Zhang F. (2006). Nutritional and osmotic roles of nitrate in a euhalophyte and a xerophyte in saline conditions. New Phytol..

[29] Kudo N., Fujiyama H. (2010). Responses of halophyte Salicornia bigelovii to different forms of nitrogen source. Pedosphere.

[30] Yuan J.F., Feng G., Ma H.Y., Tian C.Y. (2010). Effect of nitrate on root development and nitrogen uptake of Suaeda physophora under NaCl salinity. Pedosphere.

[31] Niu X., Bressan R.A., Hasegawa P.M., Pardo J.M. (1995). Ion homeostasis in NaCI stress environments. Plant Physiol..

[32] Barbier-Brygoo H., Vinauger M., Colcombet J., Ephritikhine G., Frachisse J.M., Maurel C. (2000). Anion channels in higher plants: Functional characterization, molecular structure and physiological role. Biochim. Biophys. Acta Biomembr..

[33] Zifarelli G., Pusch M. (2010). CLC transport proteins in plants. FEBS Lett..

[34] Nedelyaeva O.I., Shuvalov A.V., Balnokin Y.V. (2020). Chloride Channels and Transporters of the CLC Family in Plants. Russ. J. Plant Physiol..

[35] Jentsch T.J., Friedrich T., Schriever A., Yamada H. (1999). The CLC chloride channel family. Pflugers Arch. Eur. J. Physiol..

[36] Miller C. (2006). ClC chloride channels viewed through a transporter lens. Nature.

[37] Marmagne A., Vinauger-Douard M., Monachello D., De Longevialle A.F., Charon C., Allot M., Rappaport F., Wollman F.A., Barbier-Brygoo H., Ephritikhine G. (2007). Two members of the Arabidopsis CLC (chloride channel) family, AtCLCe and AtCLCf, are associated with thylakoid and golgi membranes, respectively. J. Exp. Bot..

[38] Barbier-Brygoo H., De Angeli A., Filleur S., Frachisse J.-M., Gambale F., Thomine S., Wege S. (2011). Anion channels/transporters in plants: From molecular bases to regulatory networks. Annu. Rev. Plant Biol..

[39] De Angeli A., Monachello D., Ephritikhine G., Frachisse J.M., Thomine S., Gambale F., Barbier-Brygoo H. (2006). The nitrate/proton antiporter AtCLCa mediates nitrate accumulation in plant vacuoles. Nature.

[40] Isayenkov S., Isner J.C., Maathuis F.J.M. (2010). Vacuolar ion channels: Roles in plant nutrition and signalling. FEBS Lett..

[41] Wege S., De Angeli A., Droillard M.J., Kroniewicz L., Merlot S., Cornu D., Gambale F., Martinoia E., Barbier-Brygoo H., Thomine S. (2014). Phosphorylation of the vacuolar anion exchanger AtCLCa is required for the stomatal response to abscisic acid. Sci. Signal..

[42] Jentsch T.J., Pusch M. (2018). CLC chloride channels and transporters: Structure, function, physiology, and disease. Physiol. Rev..

[43] von der Fecht-Bartenbach J., Bogner M., Krebs M., Stierhof Y.D., Schumacher K., Ludewig U. (2007). Function of the anion transporter AtCLC-d in the *trans*-Golgi network. Plant J..

[44] Guo W., Zuo Z., Cheng X., Sun J., Li H., Li L., Qiu J.L. (2014). The chloride channel family gene CLCd negatively regulates pathogen-associated molecular pattern (PAMP)-triggered immunity in Arabidopsis. J. Exp. Bot..

[45] Scholl S., Hillmer S., Krebs M., Schumacher K. (2021). ClCd and ClCf act redundantly at the trans—Golgi network/early endosome and prevent acidification of the Golgi stack. J. Cell Sci..

[46] Nguyen C.T., Agorio A., Jossier M., Depré S., Thomine S., Filleur S. (2015). Characterization of the chloride channel-like, AtCLCg, involved in chloride tolerance in Arabidopsis thaliana. Plant. Cell Physiol..

[47] Ashraf M., Akram N.A. (2009). Improving salinity tolerance of plants through conventional breeding and genetic engineering: An analytical comparison. Biotechnol. Adv..

[48] Volkov V. (2015). Salinity tolerance in plants. Quantitative approach to ion transport starting from halophytes and stepping to genetic and protein engineering for manipulating ion fluxes. Front. Plant Sci..

[49] Mishra A., Tanna B. (2017). Halophytes: Potential resources for salt stress tolerance genes and promoters. Front. Plant Sci..

[50] Jha R., Patel J., Mishra A., Jha B., Hasanuzzaman M., Shabala S., Fujita M. (2019). Introgression of halophytic salt stress-responsive genes for developing stress tolerance in crop plants. Halophytes and Climate Change: Adaptive Mechanisms and Potential Uses.

[51] Nedelyaeva O.I., Shuvalov A.V., Mayorova O.V., Yurchenko A.A., Popova L.G., Balnokin Y.V., Karpichev I.V. (2018). Cloning and functional analysis of SaCLCc1, a gene belonging to the chloride channel family (CLC), from the halophyte *Suaeda altissima* (L.) Pall. Dokl. Biochem. Biophys..

[52] Nedelyaeva O.I., Shuvalov A.V., Karpichev I.V., Beliaev D.V., Myasoedov N.A., Khalilova L.A., Khramov D.E., Popova L.G., Balnokin Y.V. (2019). Molecular cloning and characterisation of SaCLCa1, a novel protein of the chloride channel (CLC) family from the halophyte *Suaeda altissima* (L.) Pall. J. Plant Physiol..

[53] López-Rodríguez A., Cárabez Trejo A., Coyne L., Halliwell R.F., Miledi R., Martínez-Torres A. (2007). The product of the gene GEF1 of Saccharomyces cerevisiae transports Cl—across the plasma membrane. FEMS Yeast Res..

[54] Robinson S.P., Downton W.J.S. (1985). Potassium, sodium and chloride ion concentrations in leaves and isolated chloroplasts of the halophyte Suaeda australis R. Br. Aust. J. Plant Physiol..

[55] Yuorieva N.O., Voronkov A.S., Tereshonok D.V., Osipova E.S., Platonova E.V., Belyaev D.V. (2018). An assay for express screening of potato transformants by GFP fluorescence. Mosc. Univ. Biol. Sci. Bull..

[56] Shuvalov A.V., Yurchenko A.A., Nedelyaeva O.I., Myasoedov N.A., Karpichev I.V., Khalilova L.A., Popova L.G., Balnokin Y.V. (2021). Identification of Some Anion Transporter Genes in the Halophyte *Suaeda altissima* (L.) Pall. and Their Expression under Nitrate Deficiency and Salinity. Russ. J. Plant Physiol..

[57] Eldarov M.A., Baranov M.V., Dumina M.V., Shgun A.A., Andreeva N.A., Trilisenko L.V., Kulakovskaya T.V., Ryasanova L.P., Kulaev I.S. (2013). Polyphosphates and exopolyphosphatase activities in the yeast Saccharomyces cerevisiae under overexpression of homologous and heterologous PPN1 genes. Biochemistry.

[58] Sambrook J., Fritsch E.F., Maniatis T. (1989). Molecular Cloning: A Laboratory Manual.

[59] Gaxiola R.A., Yuan D.S., Klausner R.D., Fink G.R. (1998). The yeast CLC chloride channel functions in cation homeostasis. Proc. Natl. Acad. Sci. USA.

[60] Sherman F., Christine G., Gerald R.F. (1991). Getting started with yeast. Guide to Yeast Genetics and Molecular Biology.

[61] Jones D.T., Taylor W.R., Thornton J.M. (1992). The rapid generation of mutation data matrices from sequences. Bioinformatics.

[62] Hechenberger M., Schwappach B., Fischer W.N., Frommer W.B., Jentsch T.J., Steinmeyer K. (1996). A family of putative chloride channels from Arabidopsis and functional complementation of a yeast strain with a CLC gene disruption. J. Biol. Chem..

[63] Lurin C., Geelen D., Barbier-Brygoo H., Guern J., Maurel C. (1996). Cloning and functional expression of a plant voltage-dependent chloride channel. Plant Cell.

[64] Jentsch T.J., Steinmeyer K., Schwarz G. (1990). Primary structure of Torpedo marmorata chloride channel isolated by expression cloning in Xenopus oocytes. Nature.

[65] Jentsch T.J. (2008). CLC chloride channels and transporters: From genes to protein structure, pathology and physiology. Crit. Rev. Biochem. Mol. Biol..

[66] Jentsch T.J. (2015). Discovery of CLC transport proteins: Cloning, structure, function and pathophysiology. J. Physiol..

[67] Dutzler R. (2004). The structural basis of ClC chloride channel function. Trends Neurosci..

[68] Zifarelli G., Pusch M. (2009). Conversion of the 2 Cl^−^/1 H^+^ antiporter ClC-5 in a NO_3_^−^/H^+^ antiporter by a single point mutation. EMBO J..

[69] Wege S., Jossier M., Filleur S., Thomine S., Barbier-Brygoo H., Gambale F., De Angeli A. (2010). The proline 160 in the selectivity filter of the Arabidopsis NO_3_^−^/H^+^ exchanger AtCLCa is essential for nitrate accumulation in planta. Plant J..

[70] Waterhouse A.M., Procter J.B., Martin D.M.A., Clamp M., Barton G.J. (2009). Jalview Version 2—A multiple sequence alignment editor and analysis workbench. Bioinformatics.

[71] Bergsdorf E.Y., Zdebik A.A., Jentsch T.J. (2009). Residues important for nitrate/proton coupling in plant and mammalian CLC transporters. J. Biol. Chem..

[72] Accardi A., Walden M., Nguitragool W., Jayaram H., Williams C., Miller C. (2005). Separate ion pathways in a Cl^−^/H^+^ exchanger. J. Gen. Physiol..

[73] Miyazaki H., Uchida S., Takei Y., Hirano T., Marumo F., Sasaki S. (1999). Molecular cloning of CLC chloride channels in Oreochromis mossambicus and their functional complementation of yeast CLC gene mutant. Biochem. Biophys. Res. Commun..

[74] Kida Y., Uchida S., Miyazaki H., Sasaki S., Marumo F. (2001). Localization of mouse CLC-6 and CLC-7 mRNA and their functional complementation of yeast CLC gene mutant. Histochem. Cell Biol..

[75] Nakamura A., Fukuda A., Sakai S., Tanaka Y. (2006). Molecular cloning, functional expression and subcellular localization of two putative vacuolar voltage-gated chloride channels in rice (*Oryza sativa* L.). Plant Cell Physiol..

[76] Wei P., Che B., Shen L., Cui Y., Wu S., Cheng C., Liu F., Li M.W., Yu B., Lam H.M. (2019). Identification and functional characterization of the chloride channel gene, GsCLC-c2 from wild soybean. BMC Plant Biol..

[77] Accardi A. (2015). Structure and gating of CLC channels and exchangers. J. Physiol..

[78] Lv Q.D., Tang R., Liu H., Gao X.S., Li Y.Z., Zheng H.Q., Zhang H.X. (2009). Cloning and molecular analyses of the Arabidopsis thaliana chloride channel gene family. Plant Sci..

[79] Flowers T.J., Troke P.F., Yeo A.R. (1977). The Mechanism of Salt Tolerance in Halophytes. Annu. Rev. Plant Physiol..

[80] Flowers T.J., Colmer T.D. (2015). Plant salt tolerance: Adaptations in halophytes. Ann. Bot..

[81] Balnokin Y.V., Kotov A.A., Myasoedov N.A., Khailova G.F., Kurkova E.B., Lun’kov R.V., Kotova L.M. (2005). Involvement of long-distance Na^+^ transport in maintaining water potential gradient in the medium-root-leaf system of a halophyte *Suaeda altissima*. Russ. J. Plant Physiol..

