# Supplementary material for: Molecular Cloning and Characterization of SaCLCd, SaCLCf, and SaCLCg, Novel Proteins of the Chloride Channel Family (CLC) from the Halophyte Suaeda altissima (L.) Pall
Source: Plants (Basel). 2022 Feb 2;11(3):409. doi: 10.3390/plants11030409 (PMC8839641; doi:10.3390/plants11030409)
Supplement: Supplementary file 1 [file plants-11-00409-s001.zip › plants-1556168 -supplementary/plants-1556168-supplementary.pdf]

Table S1. List of the primers used in the study.

Primer designation	Primer name	Nucleotide sequences (5´-3´)
amplification of the partial cDNA fragments	SaCLCdF	5´GGGCACTATTCAATCAGCTT3´
	SaCLCdR	5´CCTTTCGCGTAATTAGTCC3´
	SaCLCgF	5´TTCCATGGTTTGCACCTT3´
	SaCLCgR	5´CCCCTTCCCAAAATCCAC3´
	SaCLCfF	5´GTATCAAATGAATTACTCGGGAA3´
	SaCLCfR	5´CGTTCGCAGTTTCACCA3´
amplification of the 3´- and 5´-end fragments (3´- and 5´-RACE)	SaCLCd_5'RACE_R3	5´ACTTCTGATTCTGGGCAAGG3´
	SaCLCd_5'RACE_R1	5´CGATTATTTTAACTCGTGCACCT3´
	SaCLCd_5'RACE_R2	5´CTCGTGCACCTTTCTTGTGCA3´
	SaCLCg_5'RACE_R2	5´ACTGGCAAGATCATTGTAGTGTCCA3´
	SaCLCg_5'RACE_R3	5´GGGACACTGGAATTTTTTGAAGTTACC3´
	SaCLCg_5'RACE_R1	5´CAAGCCTCAGAAGCATCACCAG3´
	SaCLCf_5'RACE_R4	5´CTCCGCACAGCATACCCAAAAT3´
	SaCLCf_5'RACE_R2	5´GTACAATGGCAGTTCTGCTGCT3´
	SaCLCf_5'RACE_R3	5´GACGGCACAGTAAAAGCTGATTTAT3´
	SaCLCd_3'RACE_F1	5´GATAGATCACACCAGGAGCGGA3´
	SaCLCd_3'RACE_F2	5´GGAGCGGAGAGACACTTGTT3´
	SaCLCd_3'RACE_F3	5´TGCCTAGTGATTCAAGAGGTGG3´
	SaCLCg_3'RACE_F1	5´CTTCGAGCCCATCTTGTTGTATTGCT3´
	SaCLCg_3'RACE_F2	5´GCATTTGAGCAGTTCTCTTCTGTGGA3´
	SaCLCf_3'RACE_F2	5´ATGCTCAAGACACTGGAGGC3´
	SaCLCf_3'RACE_F3	5´GAGAGATGCAATGAAATTTATGCGTGA3´
	SaCLCf_3'RACE_F1	5´GCTCTGGTGGTTGACGATAAGAATCT3´
amplification of the full size cDNAs	pMB_SaCLCd_F	5´ACACACATAAACAAACCATGCTGTCGAATCATTTCCAGAAT3´
	pMB_SaCLCd_R	5´ATCGATACCGTCGACCTCGAGCTATAAATTATTTTGCATCAGAAGACCAT3´

	pMB_SaCLCg_F	5′ACACACATAAACAAACCATGGCA CCCCAATTCAATGGAGGT3′
	pMB_SaCLCg_R	5′ATCGATACCGTCGACCTCGAGCTA GAAGAATTTAGCAAAGGGCGAAAG CT3′
	pMB1_SaCLCf_F	5′ACACACATAAACAAACCATGATG CATGGTTTACTTGAAATTTTG3′
	pMB1_SaCLCf_R	5′ATCGATACCGTCGACCTCGAGTTA GTGGCCATTGACGATCATTTCCT3′
qRT-PCR	SaClCd_F1	5′GGCTTCCCTGTGATAGATC3′
	SaClCd_R1	5′CACCTCTTGAATCACTAGGC3′
	SaClCg_F1	5′GGCTCCATGAGAATGACT3′
	SaClCg_R1	5′TGAAACCATCAGCCACG3′
	SaClCf_F1	5′GAAATGCTGCTGTTGCC3′
	SaClCf_R1	5′CAGAAGCAGAACAGATGTCA3′
	SaAct7_F1	5′AGATTCCGTTGCCCAG3′
	SaAct7_R1	5′ATTTCCTTGCTCATACGGTCA3′
	SaeEF1alfa_F1	5′TGAGATGTGTGGCAATCC3′
	SaeEF1alfa_R1	5′GTTGCTTCTGACTCCAAGAAT3′
amplification of the linear pMB1 form	pMB1_F	5′CATGGTTTGTTTATGTGTGTTTATT C3′
	pMB1_R	5′CTCGAGGTCGACGGTATCGATAAG C3′

Table S2. Conserved amino acids motifs and residues in SaCLCd, SaCLCf and SaCLCg sequences and their coordinates.

	SaCLCd	SaCLCf	SaCLCg
Gating Glu	E 190	E 55	–
Proton Glu	E 257	–	E 262
(1) residues in the selectivity filter, a.a.	G**S**GIPE 146–151	S**S**KSSQ 18–23	G**S**GIPE 151–156
(2) GKxGPxxH, a.a.	188–195	53–60	193–200
(3) PxxGxLF, a.a.	247–253	99–105	252–258
CBS1, a.a.	589–641	427–475	588–641
CBS2, a.a.	703–751	729–779	699–750
