# Supplementary figures and images for: Molecular Cloning and Characterization of SaCLCd, SaCLCf, and SaCLCg, Novel Proteins of the Chloride Channel Family (CLC) from the Halophyte Suaeda altissima (L.) Pall
Source: Plants (Basel). 2022 Feb 2;11(3):409. doi: 10.3390/plants11030409 (PMC8839641; doi:10.3390/plants11030409)

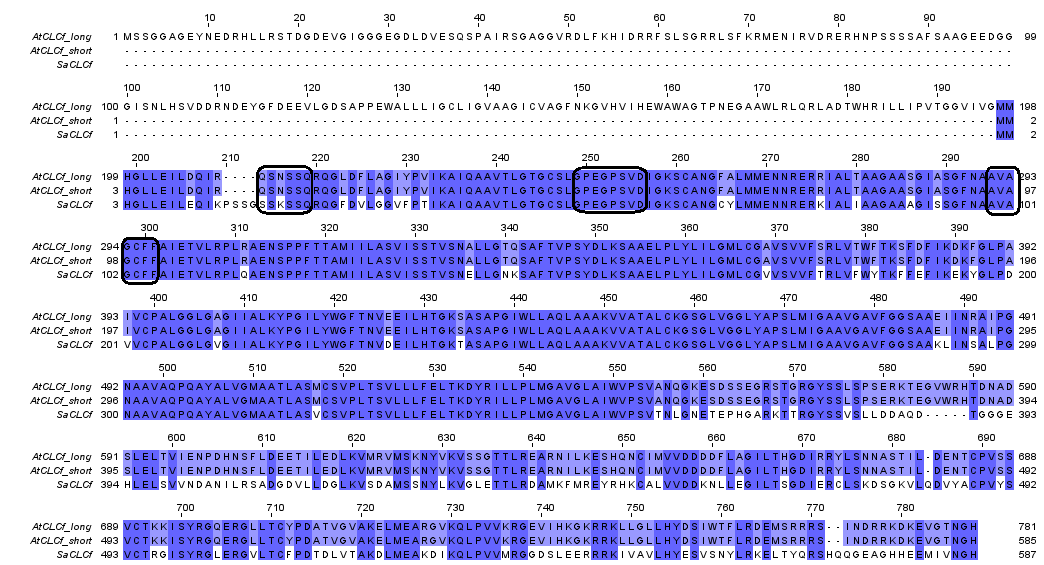

Supplement: Supplementary file 1 [file plants-11-00409-s001.zip › plants-1556168 -supplementary/plants-1556168 -Figure S1.png]
